# Specialized Pro-Resolving Lipid Mediators in Cystic Fibrosis

**DOI:** 10.3390/ijms19102865

**Published:** 2018-09-21

**Authors:** Réginald Philippe, Valerie Urbach

**Affiliations:** INSERM, U1151, Institut Necker Enfants Malades, 75993 Paris, France; reginald.philippe@inserm.fr

**Keywords:** resolvin, lipoxin, cystic fibrosis

## Abstract

In cystic fibrosis (CF), impaired airway surface hydration (ASL) and mucociliary clearance that promote chronic bacterial colonization, persistent inflammation, and progressive structural damage to the airway wall architecture are typically explained by ion transport abnormalities related to the mutation of the gene coding for the Cystic Fibrosis Transmembrane Conductance Regulator (CFTR) channel. However, the progressive and unrelenting inflammation of the CF airway begins early in life, becomes persistent, and is excessive relative to the bacterial burden. Intrinsic abnormalities of the inflammatory response in cystic fibrosis have been suggested but the mechanisms involved remain poorly understood. This review aims to give an overview of the recent advances in the understanding of the defective resolution of inflammation in CF including the abnormal production of specialized pro-resolving lipid mediators (lipoxin and resolvin) and their impact on the pathogenesis of the CF airway disease.

## 1. Cystic Fibrosis

**Figure 1:**
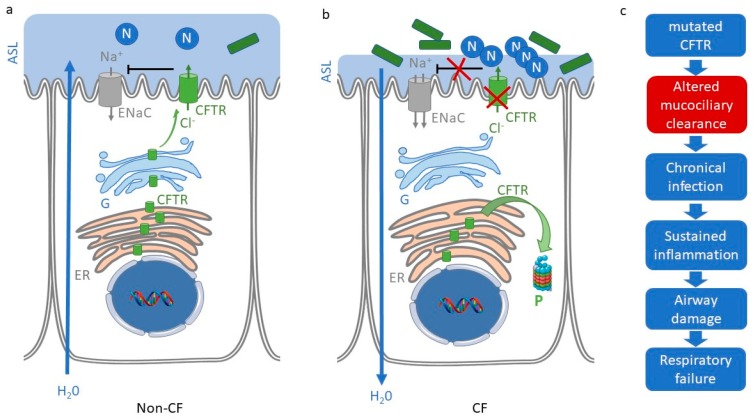
Cystic fibrosis (CF) and non-CF airway epithelial transport. (**a**) In non-CF airway epithelium, the airway surface liquid (ASL light blue) layer height is finely regulated by transepithelial chloride secretion and sodium absorption that involve the activities of the CFTR chloride channel (green arrow through CFTR) and the epithelial sodium channel (grey arrow through ENaC), respectively. In this tissue, the CFTR protein is normally expressed and trafficked (ER: endoplasmic reticulum, G: Golgi) toward the apical membrane of epithelial cells to play a role as a chloride channel and to down regulate ENaC activity. (**b**) In the case of the most common CF mutation, the CFTR protein is retained in the ER and degraded by the proteasome (P). This results in altered chloride secretion trough CFTR and regulation of ENaC (red cross) and leads to an ASL height decrease that favors bacterial (green rectangle) and neutrophil (N) accumulation in the airway. (**c**) Classical hypothesis for CF airway pathogenesis showing the central role of dehydration/ altered mucociliary clearance (red) that results from ion transport abnormality.

Cystic fibrosis (CF) is due to the mutation of the gene coding for the CFTR Chloride channel. Among more than 2000 mutations of the *cftr* gene, the most common (F508del) results in a protein folding defect, its retention in the endoplasmic reticulum (ER), and degradation by the proteasome [1]. CF affects many organs where the CFTR protein is normally expressed, however, the progressive lung destruction is the main cause of morbidity and mortality. In the healthy lung, mucociliary clearance, which requires optimal hydration of the airway surface liquid (ASL), propels inhaled particles, micro-organisms, and allergens towards the pharynx so that they can be expectorated and removed from the lungs, thus protecting from infection and inflammation. Mutations of *cftr* result in defective Cl^−^ secretion and Na^+^ hyperabsorption in airway epithelium. This ion transport dysfunction contributes to a reduction of the periciliary fluid volume and impairs mucociliary clearance, as seen in Figure 1, thus favoring bacterial colonization and sustained inflammation.

The focus on the function of CFTR in regulating epithelial ion transport and airway surface hydration provided a convincing explanation of the pathogenesis of airway disease in CF. The discovery in the CF epithelium of reduced ASL height and poor mucociliary clearance [2] was a significant step towards understanding this process in greater detail, as shown in Figure 1. However, the studies of altered ion and water transport alone failed to fully elucidate the mechanism by which the *cftr* gene mutation leads to pathogenesis in the CF lung. Furthermore, evidence began to emerge in the mid-1990s that airway inflammation begins much earlier than had previously been appreciated, even in children who are asymptomatic at the time of testing [3,4]. Overall, despite decades of research, the precise pathogenesis of the lung disease in CF remains not well understood.

## 2. Persistent Inflammation in Cystic Fibrosis

The acute inflammatory response is a normal host-protective response to contain foreign invaders. In CF, the inflammatory reaction is characterized by a predominant neutrophilic component and increased concentrations of pro-inflammatory mediators, including TNF-α, IL-1β, IL-6, IL-8, IL-17, IL-33, GM-CSF, G-CSF, and HMGB-1 [5]. An excessive airway inflammation is developed in early life of patients with CF and persists even in absence of infection [3,6,7].

Two main factors lead to sustained airway inflammation: an increased neutrophil recruitment and their defective elimination (for recent reviews Chmiel et al. [5,8]). Recurrent infections result in neutrophil over-recruitment in CF patients’ airways. In addition, it has been proposed that the excess of pro-inflammatory mediators’ production by airway epithelial cells, which triggers neutrophil recruitment, is related to a CFTR-dependent activation of the nuclear factor kappa-light-chain-enhancer of activated B cells (NF-κB) pathway, as well as a decreased interferon-γ production [9,10,11]. Increased neutrophil count is also related to the defective mechanisms of elimination related to the altered airway mucociliary clearance resulting from CFTR loss of function and Na^+^ hyper-absorption, as seen in Figure 1. Furthermore, neutrophils from CF patients are less sensitive to apoptosis and CF macrophages show a decreased phagocytic activity [12,13,14,15].

Neutrophils also present functional defects. The loss of bacteria-killing capacity of CF neutrophils contributes to the maintenance of infection and persistence of inflammation [16,17]. The altered efferocytosis and mucociliary clearance result in the necrosis of neutrophils in the airways. This results in the liberation of toxic and pro-inflammatory compounds and to the recruitment of new neutrophils, leading to the persistence of inflammation [18,19].

Overall, it does not appear that the excessive inflammatory response in CF results from altered triggering mechanisms but is mainly due to its inefficiency to contain infection and to abnormality in its resolution phase [5,8].

## 3. Resolution of Inflammation

The acute inflammatory response is normally self-limited with an active resolution phase designed to restore tissue homeostasis. The resolution of inflammation was initially thought to be a passive process and that inflammatory mediators from the initiation of the acute response (e.g., chemoattractants, complement components, prostaglandins, chemokines and cytokines) would simply dilute and dissipate to stop the infiltration of leukocytes into the tissues. However, studies performed by Serhan and colleagues on mouse inflammatory exudates revealed that acute inflammatory responses involve an active resolution phase carried out by specialized pro-resolving eicosanoid mediators (SPM) such as Lipoxins (LX) [20], resolvins (Rv) [21], protectins (PD) [22], and maresins (Mar) [23] that are locally produced from polyunsaturated fatty acids.

The temporal evolution of acute inflammation and its active resolution involves the sequential biosynthesis and activity of characteristic classes of an eicosanoid mediator in a process termed “class switching” [24]. Leukotriene B_4_ (LTB_4_) plays its role in amplification and propagation of inflammation [24] acting in concert with the peptide Interleukin 8 (IL8) as a potent neutrophil chemo-attractant [25,26]. Both LTB_4_ and IL8 are negatively correlated with pulmonary function in CF. Lipoxin A_4_ (LXA_4_) is the first eicosanoid mediator to be expressed in the active resolution phase and down-regulates neutrophil effector functions: limiting neutrophil chemotaxis, adherence and transmigration, and inhibiting superoxide anion and peroxynitrite generation [27]. Leukotrienes and lipoxins are closely related metabolites of arachidonic acid and can be synthesised from a common unstable intermediate [25]. They exhibit counter-regulatory effector functions and LXA_4_ inhibits LTB_4_ induced neutrophil transmigration in a dose dependent manner [28]. Along with lipoxins, other endogenous lipid mediators, including omega-3-derived resolvins, protectins, and maresins, stimulate and promote resolution of inflammation, clearance of microbes, tissue regeneration, and pain reduction, but do not evoke unwanted immunosuppression. SPMs are also involved in the regulation of adaptive immunity of B and T lymphocytes and can lower the amount of antibiotics needed to clear infections and reduce the potential for antibiotic resistance [29]. The abnormal process of resolution of inflammation is now considered a pathophysiologic basis associated with widely occurring diseases such as cardiovascular disease, neurodegenerative diseases, asthma, diabetes, and obesity, as well as arthritis, periodontal diseases, and cystic fibrosis.

## 4. Abnormalities of Lipid Levels in Cystic Fibrosis

Before the gene defect responsible for CF was identified, it was suggested that fatty acid metabolism abnormalities were responsible for the clinical symptoms of the CF disease. In 1962 Kuo et al. proposed that the disease was characterized by low linoleic acid (LA) concentrations in plasma and tissues [30]. Several groups also reported an important unbalance of unsaturated fatty acid with increased release of arachidonic acid (AA) and decreased levels of docosahexaenoic acid (DHA) in CF [31,32,33]. These lipid abnormalities were mainly explained by the fat malabsorption caused by the pancreatic insufficiency present in 85% of the patients [34,35]. In addition, CFTR dysfunction has been related to increased expression and activity of phospholipase A2 (PLA2) [36,37,38,39,40,41]. Since the release of AA by phospholipases is rate-limiting for the eicosanoid synthesis, an abnormal activity of PLA2 is a possible mechanism involved in increased AA levels in CF [42]. Increased oxidative stress, which is well documented in CF, has been also suggested as a cause of the fatty acid abnormalities seen in CF [43,44]. However, several studies were performed with supplementation of DHA (that should correct the unbalance between AA and DHA) to patients with CF, and failed to show significant clinical improvement [45,46,47].

A few groups have studied the levels of lipoxin, a metabolite of AA that could play a role in the pathophysiology of CF airway disease [48,49]. The mean content in LXA4, either absolute or adjusted to neutrophil count, did not significantly change in bronchoalveolar lavage (BAL) samples from patients with CF when compared to BAL samples from individuals without CF [48,50]. However, a significant decrease in LXA4 levels adjusted to neutrophil count was found in BAL from patients with CF when compared to BAL from patients with an airway inflammation but without CF [49]. Furthermore, a significant decrease in LXA4 levels adjusted to the pro-inflammatory cytokine IL8 (LXA4/IL8 ratio) was found in BAL samples of CF patients compared to control subjects [48,49]. In BAL and sputum samples from patients with CF, inter-individual changes in LXA4 content did not correlate with IL8 content [48]. The abnormality of eicosanoid class switching has been further investigated by analyzing LXA4/LTB4 ratios in BAL samples. In individuals without CF, LXA4/LTB4 ratio was related to infection, and was significantly higher in sterile samples than that measured in BAL samples from which pathogens were cultured. In contrast, in BAL samples from patients with CF, LXA4/LTB4 ratio did not vary with infection status and was uniformly depressed when compared with BAL from control children [48]. Consistent with reduced levels of DHA, RvD1/Il8 was also found to be decrease in sputa from CF patients, even in the absence of an acute infection or exacerbation [51]. Taken together, these data demonstrated that abnormalities in lipid metabolism, including SPM biosynthesis, characterize the CF airway disease.

## 5. Cellular Mechanisms Involved in the Defective Production of SPM in Cystic Fibrosis

**Figure 2:**
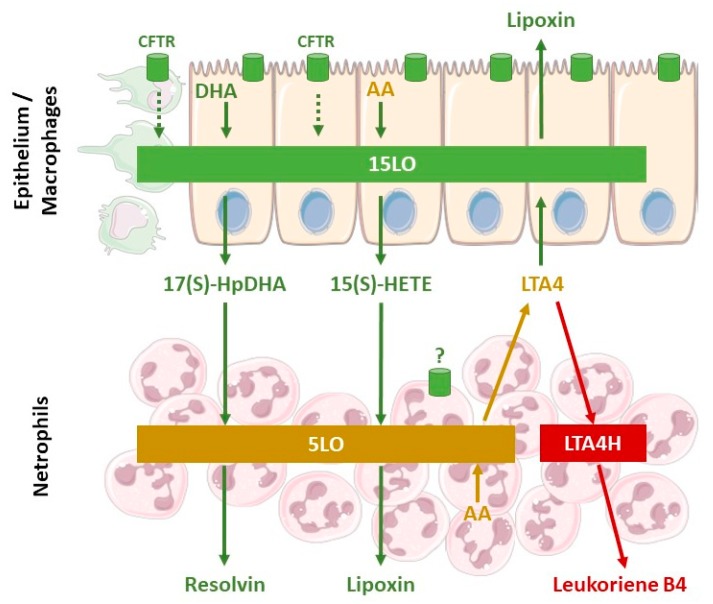
Schematic view of specialized pro-resolving eicosanoid mediators (SPM) biosynthesis pathways. Lipoxin and resolvin result from lipoxygenases interactions. Transcellular biosynthesis can occur via the interactions of two or more cell types expressing different enzymes. Epithelial cells and macrophages express the 15-lipoxygenase (15LO), that metabolizes docosahexaenoic acid (DHA) and arachidonic acid (AA) to produce 17(S)-HpDHA and 15-Hydroxyeicosatetraenoic acid (15(S)-HETE), respectively. These products of 15LO activity are the substrates for the 5LO expressed in neutrophils, to synthetize resolvin and lipoxin (pro-resolving mediators). The 5LO can also metabolize AA into leukotriene A4 (LTA4), which can be substrate of both, the 15LO to produce lipoxin or the LTA4H to produce leukotriene B4 (pro-inflammatory mediator). The pro-resolving pathways are illustrated in green, the pro-inflammatory pathway in red, and enzyme/product/substrate involved in both pathways in brown. The precise mechanism by which CFTR could affect LO activity is not known (green dotted arrow).

To date, approximately 30 kinds of SPMs have been found in inflammatory exudates: lipoxins, D-series and E-series resolvins, protectins, and maresins. They are produced at inflammatory sites from essential fatty acids (AA, DHA and eicosapentaenoic acid (EPA)) by the interaction of lipoxygenase (LO) activities within several cell types including leukocytes, platelets and epithelium, as seen in Figure 2 [25,52]. The level of expression of 15LO in airway epithelial cells and macrophages and the levels of 12LO in platelets play a central role in the eicosanoid class-switching from leukotrienes B4 (LTB4), a pro-inflammatory lipid mediator, toward SPMs. Indeed, the activity of 15LO favors LXA4 (pro-resolving) synthesis at the expense of LTB4 (pro-inflammatory) synthesis that involves leukotriene A4 hydrolase (LTA4H) activity (Figure 2) [25]. Cellular location of 5LO also plays a crucial role in LTB4/SPM equilibrium. Inhibition of the CaM kinases by RvD1 favors extra-nuclear location of 5LO, and LXA4 biosynthesis at the expense of LTB4 [53]. A reduced expression of 12/15LO in the airway of a CF mouse model as well as a reduced 15LO2 level in the BAL of patients with F508del mutation was described [48,49]. A defective 12LO activity has been reported in platelets during CFTR inhibition [54]. The 15LO expression was shown to be decreased by 50% in nasal epithelium of patients with CF compared to non-CF [55]. The 5LO was initially thought to be restricted to leucocytes, but is also expressed in human bronchial epithelial cells [56]. The cellular mechanism by which CFTR could affect 5LO, 12LO, 15LO, 15LO2 and/or LTA4H level of expression, cellular localization and activities in CF airways remains unclear. However, several data suggested that the abnormal SPM production in CF is related to abnormal LO expression and/or activity in cells that fail to normally express CFTR, including CF airway epithelium and platelets [48,49,54,55].

## 6. SPM Impact on CF Airway Epithelial Transport and ASL Height

In CF, the decreased levels in ASL height has been reported to be mainly due to a defect in trafficking and function of CFTR as a Cl^−^ channel as well as its impact on ENaC regulation leading to an increase in Na^+^ absorption (Figure 2).

The calcium-activated chloride channel TMEM16A (Transmembrane member 16A) and the SLC26A9 (Solute Carrier Family 26 Member 9) are two other anion channels that provide the critical regulation of mucus hydration of airway epithelium [57]. Both TMEM16A and SLC26A9 activities which attenuate airway inflammation and prevent mucus obstruction during airway inflammation have been considered as alternative therapeutic targets to bypass CFTR dysfunction in the airway epithelia of CF patients However, strategies for increasing intracellular calcium concentration to stimulate calcium-activated chloride secretion have been plagued by the amplification of the calcium dependent pro-inflammatory response [58,59]. Therefore, research for pharmacological long-lasting stimulation of TMEM16A independent of intracellular Ca^2+^ has been intensified [60]. The SLC26A9, a member of the solute carrier 26 family of anion transporters was shown to act as a modifier of CF lung disease severity and the response to CFTR modulator therapy [61,62]. An alternative strategy to deliver artificial non-toxic Cl^−^ transporters to CF epithelia have emerged [60].

**Figure 3:**
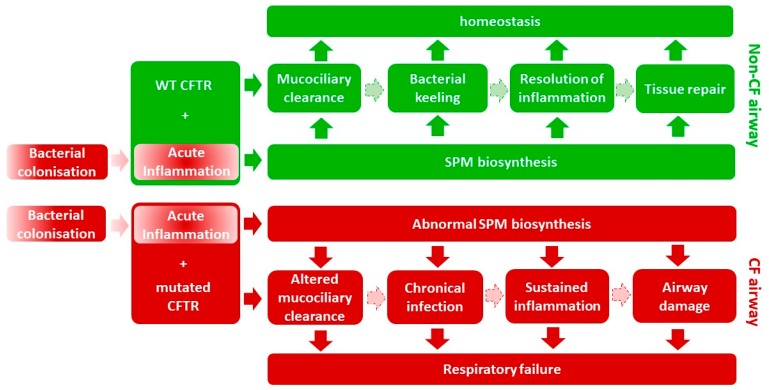
Novel hypothetical model for cystic fibrosis (CF) airway pathogenesis. In CF and non-CF airway bacterial infection triggers inflammation. In non-CF cells (top), self-limited inflammation involves the biosynthesis of specialized pro-resolving eicosanoid mediators (SPM) that enhance mucociliary clearance, bacterial killing, resolution of inflammation, and tissue repair, resulting in tissue returning to homeostasis (green). In CF cells (bottom), after initiation of inflammation, the eicosanoid class switching is altered and SPM are abnormally produced. This results into a defective mucociliary clearance, sustained inflammation, inefficient bacterial killing and tissue repair that lead to respiratory failure (red). This model enhances the role of SPM in the resolution phase of inflammation compared to the impact of dehydration and altered mucociliary clearance.

On another side, consistent with their effect in resolution of inflammation and stimulation of innate immunity, LXA4 and RvD1 treatments have been shown to target epithelium and to regulate airway epithelial ion transport [63]. LXA4 stimulates CFTR-independent chloride secretion and inhibit amiloride-sensitive Na^+^ absorption resulting in an ASL height increase in human CF and non-CF airway epithelium [64,65,66,67]. This effect involves the apical ATP (adenosine triphosphate) secretion through pannexin channels induced by LXA4, leading to purino-receptor activation and further triggering of an intracellular calcium signal [63,65]. RvD1 also increases the CF ASL height of human bronchial epithelium in a calcium-dependent manner by decreasing the amiloride-sensitive Na^+^ absorption and stimulating CFTR-independent Cl^−^ secretion. In vivo studies reflect these in vitro data showing that LXA4 and RvD1 restored the nasal transepithelial potential difference in CF mice [67]. These observations, shown in Figure 3, support the concept that the abnormal production of SPM in CF contributes to the dehydration of the airway surface and the defective airway clearance.

## 7. SPMs’ Role in Bacterial Clearance and Limiting Tissue Damage 

Resolution of acute inflammation has been shown to be crucial for ensuring bacterial clearance and limiting tissue damage. A few groups investigated the protective actions of LXA4 and RvD1 in lung infection induced by *Pseudomonas aeruginosa*. Patients with CF have a predisposition to chronic colonization and infection with *P. aeruginosa*, an organism whose presence in the CF lung is associated with progressive respiratory compromise. Antibiotics are insufficiently successful, because of resistance mechanisms developed by *P. aeruginosa* [68]. *P. aeruginosa* can escape killing by the immune system and establish chronic infections that do not resolve [69,70].

Karp et al. have shown that administration of a metabolically stable LXA4 analog in a mouse model of the chronic airway inflammation and infection associated with cystic fibrosis decreased pulmonary bacterial burden and attenuated disease severity [49]. In vitro, LXA4 has been reported to delay colonization of airway epithelium by *P. aeruginosa* by enhancing the airway transepithelial electrical resistance and the expression of the tight junction protein ZO-1 at the plasma membrane in human bronchial epithelial cells [64,71]. LXA4 also stimulates airway epithelial repair (migration and proliferation) via stimulation of ATP-sensitive potassium channel and mitogen-activated protein kinase pathway [72,73].

RvD1 decreased TNFα induced IL-8 secretion and enhanced the phagocytic and bacterial killing capacity of human CF alveolar macrophages [67]. RvD1 significantly diminished bacterial growth and neutrophil infiltration during acute pneumonia caused by a clinical strain of *P. aeruginosa*. Inoculum of *P. aeruginosa*, immobilized in agar beads, resulted in persistent lung infection and non-resolving inflammation. RvD1 significantly reduced bacterial load, leukocyte infiltration, and lung tissue damage. In murine lung macrophages sorted during *P. aeruginosa* chronic infection, RvD1 regulated the expression of Toll-like receptors, downstream genes, and microRNA (miR)-21 and 155, resulting in reduced inflammatory signaling [74]. Finally, Eickmeier et al. have reported that RvD1 levels in plasma and sputum samples from patients with CF showed a positive correlation with sputum inflammatory markers. The plasma concentrations of RvD1 were ten times higher than sputum concentrations and sputum RvD1/IL-8 levels showed a positive correlation with FEV1 [51].

These data, seen in Figure 3, provide evidence for a role of SPM in acute and chronic *P. aeruginosa* pneumonia, and for its potent pro-resolution and tissue protective properties.

## 8. Conclusions

Cystic fibrosis, the most common lethal genetic disease in Western populations, is characterized by a chronic inflammation and sustained neutrophilic inflammation of the airways. The most recent reports suggest that the persistent inflammation in CF airways appeared to be due to an altered resolution phase and efficiency to contain infection rather than abnormalities in its triggering. Early studies suggested that the abnormal fatty acid metabolism in CF played a central role in the CF disease, and more recent studies demonstrated the sustained airway inflammation to be related to an abnormal eicosanoid class switching between SPM (lipoxin and resolvin) and leukotrienes. Although the precise role of CFTR mutation on SPM biosynthesis remains unclear, the role of SPM in resolving inflammation, in increasing the airway surface liquid layer, in enhancing bacterial clearance and tissue repair strongly suggest that the altered production of pro-resolving lipid mediators play a central in CF pathogenesis, as shown in Figure 3.
